# A Recurrent Silent Mutation Implicates *fecA* in Ethanol Tolerance by *Escherichia coli*

**DOI:** 10.1186/s12866-018-1180-1

**Published:** 2018-04-18

**Authors:** Katherine M. Lupino, Kymberleigh A. Romano, Matthew J. Simons, John T. Gregg, Leanna Panepinto, Ghislaine M. Cruz, Lauren Grajek, Gregory A. Caputo, Mark J. Hickman, Gregory B. Hecht

**Affiliations:** 1 Center of Mitochondrial and Epigenomic Medicine, Children’s Hospital of Philadelphia, Philadelphia, PA USA; 2 Department of Cellular & Molecular Medicine, Cleveland Clinic, Cleveland, OH USA; 3 Department of Molecular Genetics and Microbiology, Molecular and Cellular Biology, Stony Brook University, Stony Brook, NY USA; 4 Department of Microbiology, University of Pennsylvania, Philadelphia, PA USA; 5 School of Osteopathic Medicine, Rowan University, Stratford, NJ USA; 6 Department of Biomedical and Health Sciences, Rutgers University, New Brunswick, NJ USA; 7 Revlon Research Center, Edison, NJ USA; 8 Department of Chemistry & Biochemistry, Rowan University, 201 Mullica Hill Rd, Glassboro, NJ 08028 USA; 9 Department of Biological Sciences, Rowan University, 201 Mullica Hill Rd, Glassboro, NJ 08028 USA; 10 Department of Molecular & Cellular Biosciences, Rowan University, 201 Mullica Hill Rd, Glassboro, NJ 08028 USA

**Keywords:** *Escherichia coli*, FBR5, Ethanol tolerance, Membrane permeability, *fecA*, Next-generation sequencing

## Abstract

**Background:**

An issue associated with efficient bioethanol production is the fact that the desired product is toxic to the biocatalyst. Among other effects, ethanol has previously been found to influence the membrane of *E. coli* in a dose-dependent manner and induce changes in the lipid composition of the plasma membrane. We describe here the characterization of a collection of ethanol-tolerant strains derived from the ethanologenic *Escherichia coli* strain FBR5.

**Results:**

Membrane permeability assays indicate that many of the strains in the collection have alterations in membrane permeability and/or responsiveness of the membrane to environmental changes such as temperature shifts or ethanol exposure. However, analysis of the strains by gas chromatography and mass spectrometry revealed no qualitative changes in the acyl chain composition of membrane lipids in response to ethanol or temperature. To determine whether these strains contain any mutations that might contribute to ethanol tolerance or changes in membrane permeability, we sequenced the entire genome of each strain. Unexpectedly, none of the strains displayed mutations in genes known to control membrane lipid synthesis, and a few strains carried no mutations at all. Interestingly, we found that four independently-isolated strains acquired an identical C → A (V244 V) silent mutation in the ferric citrate transporter gene *fecA*. Further, we demonstrated that either a deletion of *fecA* or over-expression of *fecA* can confer increased ethanol survival, suggesting that any misregulation of *fecA* expression affects the cellular response to ethanol.

**Conclusions:**

The fact that no mutations were observed in several ethanol-tolerant strains suggested that epigenetic mechanisms play a role in *E. coli* ethanol tolerance and membrane permeability. Our data also represent the first direct phenotypic evidence that the *fecA* gene plays a role in ethanol tolerance. We propose that the recurring silent mutation may exert an effect on phenotype by altering RNA-mediated regulation of *fecA* expression.

**Electronic supplementary material:**

The online version of this article (10.1186/s12866-018-1180-1) contains supplementary material, which is available to authorized users.

## Background

Interest in alternative and renewable fuels is high as climate change and national energy security have brought about the search for a sustainable, non-fossil fuel energy source that would be both cost effective and environmentally friendly. The conversion of plant biomass into various liquid fuels such as ethanol is an area of interest in this regard. The majority of biofuels are currently made with sugar, starch, or fats derived from plants that are also used for food and feed. There is significant and warranted concern that the use of food crops for fuel may not be sustainable [1]. A long-term solution to this problem is to produce fuels from non-edible lignocellulose [1]. The use of corn stover and wheat straw in biofuel production through fermentative pathways of *Escherichia coli* (or other organisms) has numerous potential benefits, including low production cost, use of a non-consumable agriculture byproduct, space efficiency and sustainability [2].

One such *E. coli* strain pursued for fermentation, *E. coli* KO11, suffered from a genetic instability resulting in a decrease in ethanol yield over time and the inability to be used for repeated fermentation runs [3]. Due to its ability to metabolize xylose, *E. coli* mutant FMJ39x was later used as the parent strain for artificial selections designed to produce a reusable fermenter strain [4]. The enzymes lactate dehydrogenase (*ldhA*) and pyruvate formate lyase (*pfl*) were deactivated via deletion caused by chemical mutagenesis, thus artificially removing the ability of FMJ39x to perform anaerobic fermentation of pyruvate. The ability of FMJ39x to ferment pyruvate to ethanol was restored by transforming FMJ39x with the plasmid pLOI297, created using genes from *Zymomonas mobilis* [4]. The resulting strain, FBR3, showed promise as a biocatalyst due to its higher yield and stability, along with its ability to maintain pLOI297 with 97% efficiency when grown anaerobically. However, although *E. coli* FBR3 had a 90–91% conversion rate to ethanol, it was determined to ferment 30% slower than *E. coli* KO11 [5]. The inefficiency of FBR3 led to the creation of two new strains in the FBR series by transformation of *E. coli* strains DC1368 and NZN111 with pLOI297 resulting in FBR4 and FBR5, respectively [4]. The efficiency of FBR4 and FBR5 to produce ethanol was compared and it was determined that FBR5 completed fermentation 20 h earlier and produced 90% of the theoretical yield of ethanol versus 78% for FBR4.

An additional issue associated with efficient bioethanol production is the fact that the desired product is toxic to the biocatalyst (cf. [6–9]). Research has also shown a correlation between ethanol toxicity and decreased fermentation yields [10, 11]. Ethanol has previously been found to influence the membrane of *E. coli* in a dose dependent manner and to affect the lipid composition of the plasma membrane [12–14]. At low concentrations ethanol disrupts packing and increases lipid motion in the membrane while at moderate concentrations ethanol begins selectively extracting lipids [15]. It has also been proposed that increased ethanol tolerance is correlated to increased membrane fluidity.

In the work presented here, we document that FBR5 has a markedly lower tolerance for ethanol than its K-12 ancestor and that FBR5 has a number of mutational changes in genes that have been previously been implicated in ethanol tolerance and stress response. We describe the isolation of twenty independent mutant derivatives with varying abilities to grow in the presence of ethanol. The data indicate that a subset of the strains in the collection have membrane permeability alterations, but no changes were observed in their membrane lipid constituency. Genomic analysis was carried out on all twenty of the derivative strains, and some data is suggestive that epigenetic mechanisms may be contributing to the phenotypes of these strains. Particularly noteworthy is the observation that four of the strains acquired an identical silent mutation in the ferric citrate transporter gene *fecA*. We demonstrate that *E. coli* strains that carry a *fecA* deletion or that over-express *fecA* exhibit increased ethanol tolerance, and we suggest a possible role for the recurring silent mutation in our mutant strains.

## Methods

### Bacterial strains and growth

*Escherichia coli* strains used in this study are presented in Table 1. All cultures were grown in Luria Bertani (LB) medium prepared as follows: tryptone 25 g/L, yeast 5 g/L, NaCl 5 g/L; for solid medium, 1.6% *w*/*v* agar was used. Plasmids were maintained by supplementing the LB medium with antibiotics as follows: pLOI297, 100 mg/L ampicillin; pBluescript (Stratagene) 50 mg/L ampicillin; pCA24N and pCA24N + JW4251, 25 mg/L chloramphenicol.

**Table 1:** *E. coli* strains and plasmids used in this study

Strain or plasmid	Genotype or relevant characteristics^a^	Source or reference
*E. coli* K-12		
BW25113	MG1655 derivative; F^−^, *Δ(araD-araB)567 ΔlacZ4787(::rrnB-3) λ*^*−*^ *rph-1 Δ(rhaD-rhaB)568 hsdR514*	[15]
BW25113 Δ JW4251	BW25113 *ΔfecA758::kan*	[66]
FBR5	∆*pfl*::Cm *ldhA*::kan, pLOI297	[4]
MG1655	F^−^, *λ*^*−*^ *ilvG*^−^ *rfb*-50 *rph*-1	K-12 reference strain; ATCC47076
Plasmids		
pCA24N	Cm^r^ vector, IPTG-inducible promoter	[67]
pCA24N + JW4251	pCA24N derivative carrying *fecA* gene adjacent to IPTG-inducible promoter	[67]
pLOI297	Ap^r^ Tc^r^ *pdc*^+^ *adhB*^+^	[68]

^a^*Abbreviations*: *Ap* ampicillin, *Cm* chloramphenicol, *Kan* kanamycin, *Tc* tetracycline

### Mutant isolation via alcohol pressured isolation challenges

Isolation protocol was similar to that described previously [16] with the following modifications. Twenty independent FBR5 cultures were grown in 5 mL of LB broth supplemented with both 40 g/L xylose and 100 mg/L ampicillin for 24 h at 30 °C. Following the 24 h, 10 ml of LB broth with 35 g/L ethanol was added to each culture and allowed to grow for another 24 h at 30 °C. For ten of these lineages, serial dilutions were plated onto the surface of LB agar plates containing 10 g/L (1.27% *v*/v) isopropanol, 100 mg/L ampicillin and 20 g/L xylose while the other ten lineages were grown in poured media (LB broth 1 L, agar 0.35%, xylose 20 g/L, isopropanol 10 g/L (1.27% v/v), ampicillin 100 mg/L) to simulate anaerobic growing conditions. The plates were incubated at 30 °C until noticeable growth was observed; the three largest colonies of each initial culture were then selected for further enrichment. The above procedures were repeated two more times for each culture lineage at 35 g/L ethanol and then repeated three more times per concentration at 45, 55, and 60 g/L ethanol. At the end of the entire procedure, only one mutant was saved from each of the original twenty lineages. Nomenclature for resulting strains was determined by the growing condition. The ten cultures of FBR5 used in the pour plate growth procedures were named ANA-ANJ. The ten cultures of FBR5 used in the aerobic spread plate procedures were named ARK-ART.

Plasmid-free derivatives of the parent and mutant strains were made by curing the strains of the pLOI297 plasmid via successive passages in the absence of ampicillin. These derivative strains were also subsequently transformed with pBluescript, a plasmid that carries a gene encoding for β-galactosidase production. The derivatives carrying pBluescript were used in minimal inhibitory concentration (MIC) and membrane permeability experiments.

### Survival assays

Cultures were grown overnight (12–15 h) in LB broth at 37 °C. Samples were diluted 1:200 in LB broth and grown in shaker flasks to OD_600_ = 0.6 at which point (*t* = 0) a sample was removed and a viable cell count was determined by plating samples from serial dilutions onto LB plates. Immediately following the removal of the initial sample, ethanol was added to the shaker flasks. Subsequent samples were removed from the flasks at specific times and viable cell counts determined as described above. Survival assays were performed in strains not containing a plasmid to prevent ethanol production from pLOI297, thus removing ambiguity in the experiment regarding the concentration of ethanol present in the culture flask over time.

### Minimal inhibitory concentration (MIC) assay

MIC assays were done to compare bacterial growth at varying concentrations of ethanol and performed as previously described with minor modifications [17]. Overnight cultures of mutants transformed with pBluescript were grown in LB broth containing 50 μg/ml of ampicillin at 30 °C for 10–18 h. Cultures were diluted 1:500 in fresh media and grown up to OD_600_ ~ 0.4. Cultures were then diluted to 1:1 × 10^6^ CFU/ml. 90 μl of dilute cultures were then added to 10 μl of 8 different concentrations of ethanol (100, 50, 25, 12.5, 6.25, 3.125, 1.56, and 0% by volume diluted with water) in a 96 well microtiter plate resulting in exposure concentrations ranging from 0 to 10% ethanol by volume. Culture and ethanol mixtures were grown up in static conditions overnight in 30 °C incubator for 18 h and then absorbance in each well was measured at 600 nm using a Thermo Scientific Multiskan (Thermo Fisher Scientific, Waltham, MA, USA) plate reader. An OD_600_ above 0.1 after incubation was used as the threshold to indicate growth. Reported values are the average of 3–5 separate, independent experiments.

### Membrane permeability assay using β-galactosidase

Assays using β-Galactosidase and ONPG to measure the inner membrane permeability of the 20 mutant strains were performed as previously described with minor modifications [18]. Overnight cultures of strains carrying pBluescript were grown in LB broth containing 50 μg/ml of ampicillin at 30 °C or 37 °C for 10–18 h. Overnight cultures were diluted 1:100 in fresh media and grown to an OD_600_ of 0.2–0.3. 5 ml of each diluted culture was then centrifuged at 7500 rpm for 10 min in a DuPont (Wilmington, DE) Sorvall RC 5C Plus centrifuge with a DuPont Sorvall model SLA-1500 rotor. Supernatant was removed and pellets were resuspended in 5 ml of phosphate buffer solution (PBS) (10 mM phosphate, 200 mM NaCl, pH = 7.0). In a 96 well microtiter plate, 50 μl of distilled water, 10 μl of 22 mM O-nitrophenylgalactopyranoside (ONPG), and 40 μl of resuspended culture. A positive control contained 50 μl of 10 mM cetyl trimethylammonium bromide (CTAB), 10 μl of ONPG, and 40 μl of culture. In the presence of β-galactosidase, ONPG, which is typically a colorless substrate, is hydrolyzed, resulting in galactose and o-nitrophenol (ONP), which appears yellow [19]. Absorbance was measured at 420 nm with a Multiskan plate reader. Readings were taken every 5 min for 90 min with intermittent shaking between readings. The ONPG conversion rate (ONPG/min) was quantified based on concentration of ONP using Beer’s Law. Since the permeability of ONPG through the inner membrane is normally low, this assay allowed membrane permeability to be inferred from the calculated ONPG conversion rates [18] and therefore facilitated the categorization of the 20 mutant strains by relative inner membrane permeability when cultures were grown both in the presence and absence of ethanol. Relative ONPG hydrolysis was determined by calculating the slope of the first 30 min of absorbance readings into a conversion rate. Reported values are the average of 3–5 individual experiments.

ONPG permeability assays involving bacteria exposed to ethanol were completed using the same protocol as above with one modification. For ease of experimental manipulation, ethanol was added to a 4% (*v*/v) final concentration when overnights were diluted 1:100 in fresh media.

ONPG conversion rates were compared between strains grown under different temperature or ethanol-exposure conditions. Simple ratios were taken of the average conversion rate for a given strain under different conditions to obtain relative changes in permeability behavior as a result of the differing environmental conditions. These ratios were calculated for each strain to investigate the results of shifting temperature on permeability:

1 $$ \frac{{\left(\mathrm{ONPG}\frac{\mathrm{pmol}}{\min}\right)}_{0\%\mathrm{EtOH}@37{}^{\circ}\mathrm{C}}}{{\left(\mathrm{ONPG}\frac{\mathrm{pmol}}{\min}\right)}_{0\%\mathrm{EtOH}@37{}^{\circ}\mathrm{C}}} $$

or the result of exposure to 4% ethanol:

2 $$ \frac{{\left(\mathrm{ONPG}\frac{\mathrm{pmol}}{\min}\right)}_{4\%\mathrm{EtOH}@30{}^{\circ}\mathrm{C}}}{{\left(\mathrm{ONPG}\frac{\mathrm{pmol}}{\min}\right)}_{0\%\mathrm{EtOH}@30{}^{\circ}\mathrm{C}}} $$

or any combination of ethanol exposure and temperature.

### Lipid extraction and gas chromatography mass spectrometry (GC-MS) analysis

Bacterial cultures were grown as above at 30 °C or 37 °C except that in some cases cultures received ethanol to a final concentration of 4% (*v*/v) and allowed to grow for 3 h before harvesting. Lipids were extracted as described previously [20, 21]. To summarize, cultures were centrifuged and the bacterial pellet was resuspended in 2 mL H_2_O prior to flash freezing with liquid nitrogen. The frozen cells were lyophilized overnight and then resuspended in 1 mL deionized water and transferred to a beaker. Equal parts methanol and chloroform were then added to another beaker, followed by the resuspended bacteria sample and another part methanol. After allowing stirring for fifteen minutes, another part of chloroform was added, and then after two more minutes the 2 M NaCl solution was added to allow mixing for eight to ten minutes. The solution was then transferred to a 50 mL centrifuge tube and centrifuged for ten minutes at 2800 rpm. This resulted in the formation of two clear layers with a fragile white layer separating the two. The bottom layer was extracted, put into a 10 mL glass test tube, and placed under a stream of N_2_ gas to evaporate all the contents except the lipid. After evaporation 200 μL of a H_2_SO_4_, methanol, and H_2_O (30%:28%:42%) solution was added. The tube was vortexed briefly and then placed in a 100 °C heat block for ten minutes. Then 400 ml of 6 M NH_4_OH was added followed by 400 ml of hexane and vortexing. The sample was put into a microfuge tube and centrifuged at 11000 rpm for 5 min. The top layer in the tube was removed and placed in a GC-MS vial.

Samples were analyzed on an Agilent Technologies 6890 Network GC System with a 5973 Network Mass Selective Detector. The column was coated in a nonpolar polymer of dimethylpolysiloxane and had dimensions of 30 m × 0.320 mm ID × 0.250 μm thick. The GCMS method started at room temperature before ramping and holding the samples at around 220 °C. Most peaks eluted between 19 and 30 min. Peak identification was performed using the provided Agilent software database.

### Whole genome sequencing

DNA was isolated from overnight cultures using the Gentra Puregene Yeast/Bacteria kit (Qiagen). Twenty-one multiplex libraries were made using the IntegenX Apollo 324™ System. The libraries were sequenced on a single lane of an Illumina HiSeq, to a read length of 140 bases, resulting in an average fold-coverage of 400 for each strain. The reads were mapped to the *E. coli* K-12 MG1655 reference genome (GenBank: U00096.2) [22] using BWA for Illumina [23]. The resulting SAM files were converted to BAM files using SAMtools [24]. The BAM files were visualized using Integrated Genome Viewer (IGV) [25], manually searching the genomes for SNPs, deletions, insertions, and amplifications. An insertion is predicted at a genomic location with a marked decrease in read depth, which occurs when individual sequencing reads fail to span that location. An amplification is predicted at genomic intervals containing a consistent read depth of more than 1.5-times the background read depth. All mutations are described in Additional file 1: Table S1. To reduce the possibility of overlooking some genomic mutations, the sequence data were also analyzed with FreeBayes, a powerful variant-detector package [26]. No additional mutations were identified using this second method.

## Results

### Comparison of FBR5 to the ancestral K-12 strain

**Fig. 1:**
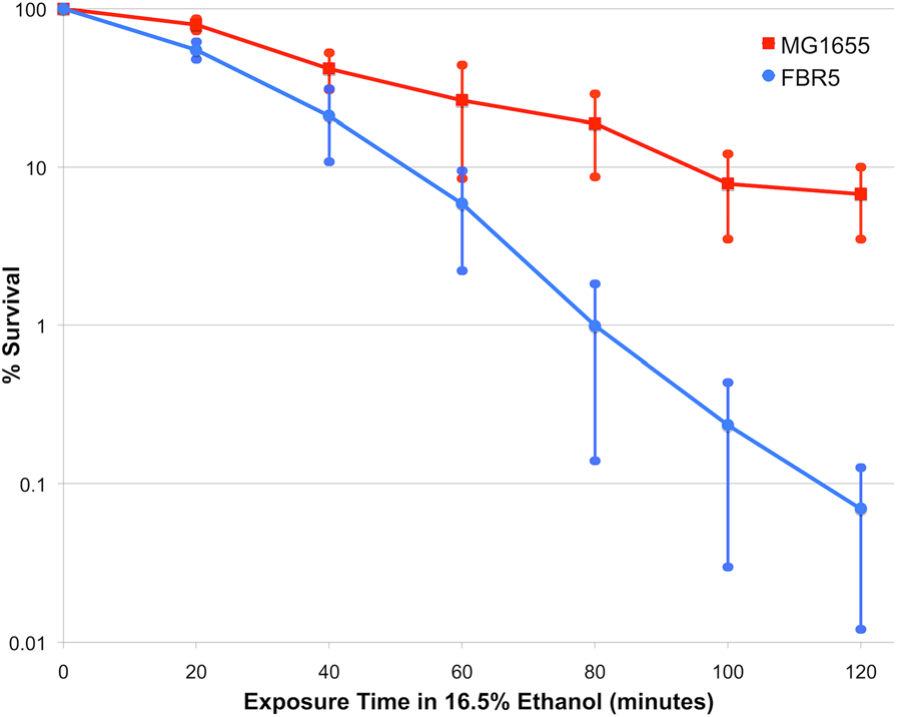
Survival curve data demonstrating that *E. coli* FBR5 has lower ethanol tolerance than its MG1655 ancestor. *E. coli* strains MG1655 and FBR5 were grown for 120 min in LB containing 16.5% (*v*/v) ethanol, and viable cell counts were conducted at specific time points during that incubation period. Each of the data points are the average of three to five independent trials. The error bars represent the Standard Error of the Mean (SEM)

**Fig. 2:**
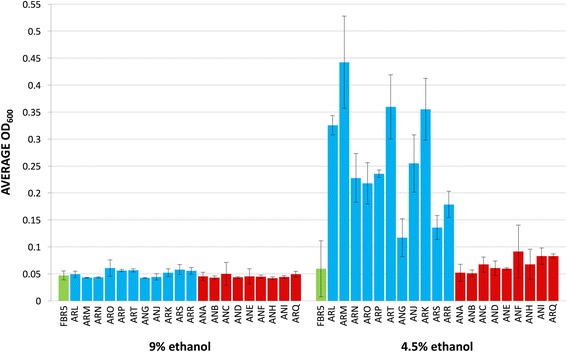
MIC experiments were conducted using serial dilutions to demonstrate acquired ethanol tolerance. Mutants were considered to have a high MIC if they were capable of showing growth above an OD of 0.1 at 600 nm at 4.5% EtOH. Strains unable to grown to above an OD of 0.1 at 600 nm at 4.5% EtOH were determined to have a low MIC. All strains, FBR5 and the 20 isolated mutants, grew to an OD under 0.1 in 9% EtOH and to an OD of ≥0.1 in 2.25% EtOH (results not shown). Data shown represents the results from at least three independent trials. The error bars represent one standard deviation above and below the mean of the data. Mutant strains are presented in the order of their MIC phenotypes and 30 °C ONPG permeability (see also Fig. 3 and Table 3). Blue columns represent data from strains categorized as “High MIC” strains and red columns represent data from strains categorized as “Low MIC” strains

Ethanol inhibition is a major limitation to the fermentative ethanol yield (cf. [6, 7, 9]). Previously described *E. coli* biocatalysts, such as the KO11 strain, have been reported as having an ethanol minimal inhibitory concentration (MIC) of roughly 4.3% *v*/v [16]. We have observed MIC values for the K-12 strains MG1655 and BW25113 at or above 5% v/v (data not shown). Because K-12 is ancestral to FBR5, this suggested that FBR5 may have comparable ethanol tolerance. However, this was not the case. In the presence of 16% (*v*/v) ethanol, FBR5 lost viability more rapidly than MG1655, exhibiting on average approximately 100-fold decreased viability at the end of a two-hour exposure (Fig. 1). In addition, MIC experiments demonstrated that FBR5 has an ethanol MIC below 4.5% v/v (Fig. 2), lower than the MIC of K-12 strains.

To understand the genetic changes that may underpin this difference in ethanol tolerance, we performed whole-genome Illumina sequencing of FBR5 and compared the genome sequence to that of the MG1655 reference genome [22]. All sequence differences are presented in Table 2. As expected from the history of the construction of FBR5 [27], we found that the strain carries an insertion in *ldhA*, as well as a 2.5 kb deletion that extends from *pflB* to the adjacent *focA* and *ycaO* genes. It is worth noting that *ldhA* has been identified previously as a gene that is upregulated in response to ethanol stress [28]. There are also point mutations in FBR5 at *gatY* and *rpoS*, two genes which have been implicated previously in alcohol tolerance and stress response [28–30]. FBR5 also carries an insertion in *gcvB*, encoding a small non-coding RNA that regulates genes involved in amino acid and oligopeptide transport [31–33]. One of these genes, *oppA*, encodes a periplasmic protein that is important for acquiring nutrient peptides and recycling peptide components of the cell wall [34, 35] and has been observed to be down regulated during the ethanol stress response [28]. A second *gcvB*-regulated gene, *dppA*, encodes a periplasmic dipeptide transport protein [36]. Although *dppA* has not been previously implicated in the ethanol stress response, a substantial number of other *dpp* genes have been observed to be downregulated in response to ethanol [28]. It is plausible that mutations in the genes highlighted above contribute to the differences in ethanol tolerance between MG1655 and FBR5.

**Table 2:** Observed mutational differences between *E. coli* strains FBR5 and MG1655^a^

Function	Gene	Mutation	Description	Notes
Ethanol pathway	***ldhA***	insertion ca. T152	D-lactate dehydrogenase	b,c
	*pflB*	5′ del up to S397	Pyruvate formate lyase	b
Cellular respiration	*dcuS*	T198I	Histidine protein kinase; regulates anaerobic fumarate metabolism in response to extracellular fumarate concentrations	
Cell wall	*mltA*	S268G	Transglycosylation of the muramic acid residue	
Membrane transport	*focA*	del	Membrane protein; formate export	b
	*gcvB*	insertion ~ nt 2,940,787	sRNA that represses ***oppA***, *dppA*, *gltI* and *livJ*	d
Metabolic functions	***gatY***	insertion ca. E149	Encodes tagatose-bisphosphate aldolase	c
Protein synthesis	*ycaO*	3′ del starting at A565	Ribosomal protein S12 methylthiotransferase accessory factor	
Transcription	*rpoA*	L300F	RNA polymerase alpha subunit	
	***rpoS***	Q33stop	RNA polymerase sigma subunit	e,f
Pseudogenes	*intQ*	F261 L		
Unknown function	*ylbE*	E39E	Domain of unknown function 1116 family member; induced by NO	
	*yphD*	possible insertion ~ V236	ABC transporter of unknown function	
Insertion sequences, transposons, and repetitive elements	*RIP321*	CG insertion at nt 4,294,403	Repetitive element	
Mutations outside of known genes		possible insertion ~ nt 2,985,196	between *yqeG* (putative hydroxy/aromatic amino acid permease) and *yqeH* (predicted LuxR family transcriptional regulator)	
		C → T at nt 3,957,957	between *ppiC* (peptidylprolyl-cis-trans isomerase; protein folding) and *yifO* (hypothetical conserved protein)	

^a^Genes previously implicated in ethanol tolerance are in boldface type. *Abbreviations*: *del* deletion, *fs* frameshift, *nt* nucleotide

^b^FBR5 carries disruptions of these genes as a consequence of its derivation from *E. coli* strain NZN111 [27]

^c^Previously implicated in ethanol stress response in [28]

^d^*OppA* and several *dpp* genes (although not *dppA*) have been previously implicated in ethanol stress response in [28]

^e^Previously implicated in ethanol tolerance in [29]

^f^Previously implicated in alcohol tolerance in [30]

### Isolation of ethanol-tolerant FBR5 mutants

Fermentation of corn fiber by FBR5 has a maximum yield of roughly 4% v/v [3], a value that is similar to the ethanol MIC that we have observed for FBR5 (Fig. 2). Thus, the development of ethanol-tolerant derivatives of FBR5 may have the potential to improve ethanol yield from corn fiber feedstock. To obtain such strains, successive alcohol isolation challenges were carried out on twenty independent cultures of FBR5 as described in the Methods. The alcohol concentrations used in our enrichment strategy were sub-lethal, and therefore the mutation rate for the appearance of ethanol tolerance could not be calculated. The 20 mutant strains resulting from this procedure were divided into two groups: ANA-ANJ for those isolated anaerobically (i.e., within pour plates) and ARK-ART for those isolated aerobically (i.e., on the surface of solid medium).

MIC experiments were used to assay differences in ethanol tolerance between the mutants and FBR5 (Fig. 2). Growth for all strains was inhibited by 9% (v/v) ethanol. At 4.5% (v/v) ethanol, growth was inhibited in many but not all mutants. Mutant strains were considered to exhibit a high MIC phenotype if, after 18 h of static incubation in 4.5% ethanol, the culture achieved an OD_600_ of 0.1 or more. Strains unable to meet this threshold were considered to have a low MIC. Nearly all of the strains in the AR group displayed a high MIC phenotype while most AN strains did not (Fig. 2). All strains surpassed an OD_600_ of 0.1 in ethanol concentrations lower than 2.25% (v/v) (data not shown).

### Inner membrane permeability assays

Previous studies have shown that ethanol exposure results in significant membrane permeability issues for microorganisms [37–39]. For *E. coli* K-12 and MG1655, it has been reported that the ratios of unsaturated and saturated lipids in the membrane do not experience large shifts in the presence of ethanol, and so the membrane fluidity increases as a result of ethanol exposure [13]. Therefore, changes in membrane fluidity and/or lipid constituents may in principle confer increased ethanol tolerance. We examined the effects of ethanol on both membrane permeability and membrane lipid composition in our mutant strains to determine the extent to which changes in membrane biology play a role in their phenotypes.

**Fig. 3:**
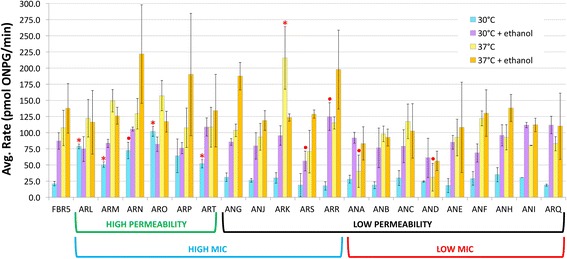
Average pmol/min conversion of ONPG was measured to determine membrane permeability of the parental FBR5 and 20 isolated mutant strains in the absence and presence of 4% ethanol at 30 °C and 37 °C. Data shown represents the results from at least three independent trials. The error bars represent one standard deviation above and below the mean of the data. Mutant strains are presented in the order of their MIC phenotypes (see Fig. 2) and 30 °C ONPG permeability; the brackets underneath the x axis correspond to the phenotype categories presented in Table 3. For each of the mutant strains, t-tests were performed to compare the results with those of FBR5 grown under the same conditions. Mutant results that are significantly different from FBR5 are indicated as a red dot (*P* < 0.05) or red asterisk (*P* < 0.01)

To determine whether the ethanol-tolerant strains have altered permeability phenotypes, we tested the ability of the substrate ONPG to diffuse across the cell membrane and be catalyzed by intracellular β-Galactosidase into a colored product (ONP) [19]. The rate of ONP production was assumed to be correlated with membrane permeability, as demonstrated previously [17]. As expected, ONP production is low in FBR5 at 30 °C (Fig. 3), indicating that membrane permeability is low under normal conditions. Some strains displayed increased ONP production at 30 °C as compared to FBR5, suggesting an increase in permeability. The 30 °C permeability data was used to sort the mutant collection as presented in Table 3. The mutants categorized as “High Permeability” exhibit greater permeability than FBR5, and strains with permeability similar to that of FBR5 were denoted as “Low Permeability” strains. Table 3 also shows that High Permeability strains consist only of mutants from the AR group, and no High Permeability strains exhibited a low MIC. Although most of the Low Permeability strains displayed a low MIC phenotype, there were several that showed a high MIC phenotype.

**Table 3:** Categorization of mutants based on 30 °C permeability and ethanol MIC

Parent Strain (Control)	High Permeability with High MIC Phenotype	Low Permeability with High MIC Phenotype	Low Permeability with High MIC Phenotype (Outlier)^a^	Low Permeability with Low MIC Phenotype
FBR5	ARL	ANG	ARR	ANA
	ARM	ANJ		ANB
	ARN	ARK		ANC
	ARO	ARS		AND
	ARP			ANE
	ART			ANF
				ANH
				ANI
				ARQ

^a^See text and Fig. 4

**Fig. 4:**
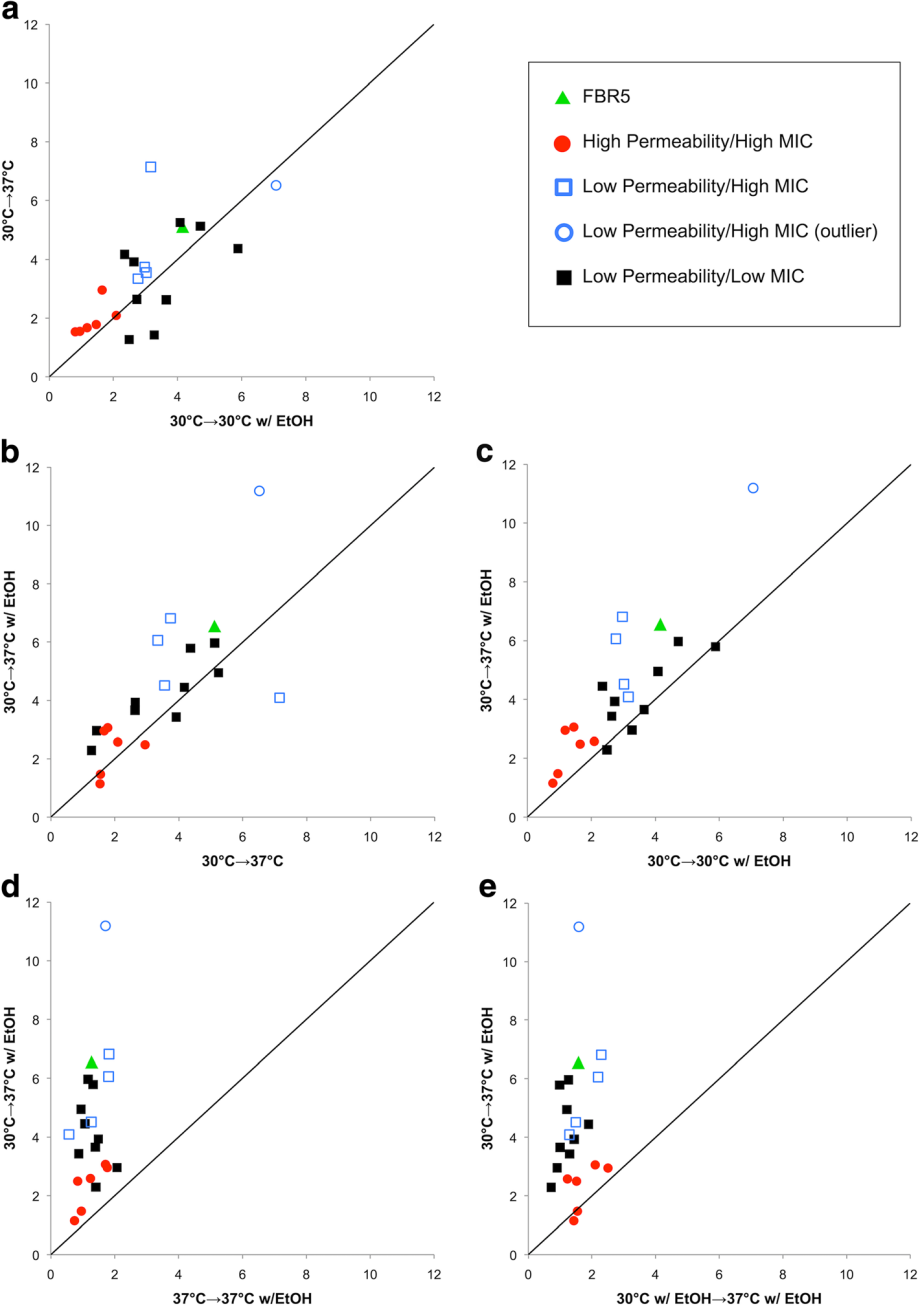
Comparisons of membrane permeability responses to environmental challenges. Symbols correspond to groups in Table 3. Ratios were calculated by comparing the average rate of uptake seen in Fig. 3 for an individual strain at the conditions indicated on the axis. Error bars are omitted for clarity. Solid black line indicates a slope of 1.0, thus representing no relative difference between the two environmental alterations indicated on the two axes. **a** Membrane permeability increase induced by ethanol exposure compared to the permeability induced by shifting culture temperature, **b** Permeability increase induced by temperature compared to the increase that results from the addition of ethanol and a temperature shift, **c** Permeability increase induced by ethanol exposure at 30 °C compared to the increase that results from the addition of ethanol and a temperature shift, **d** Effect of ethanol addition after a temperature shift and **e** Effect of temperature shift after ethanol exposure

To assess how each strain adapts to environmental stressors that are known to modulate membrane permeability, we measured ONP production by the cells following temperature shifts (30 °C to 37 °C) and the addition of 4% ethanol. FBR5 and the mutant derivatives exhibited varying changes in membrane permeability after exposure to high temperature or to ethanol. Either increasing the temperature or adding ethanol elicited an increase in permeability of roughly similar magnitude (Fig. 3). Control experiments showed no significant difference in β-galactosidase activity at 37 °C indicating the observed changes in ONPG conversion rates are governed primarily by membrane permeability (results not shown). To illustrate the relative phenotypic effect of the temperature and ethanol variables on each strain in our collection, ratios were calculated from the ONPG conversion rates shown in Fig. 3, and the ratios for each strain were then compared to one another in the plots shown in Fig. 4. For example, to determine whether the addition of ethanol at 30 °C resulted in greater relative effects as a 30 °C to 37 °C temperature shift, the ratio of ONPG conversion for each strain at 30 °C with and without ethanol were calculated as shown in Eq. 1 (see Methods) and plotted on the x axis, and the ratio for each strain at 37 °C and 30 °C were calculated as shown in Eq. 2 (see Methods) and plotted on the y axis.

In the resulting plot, shown in Fig. 4a, most of the strains cluster close to the midpoint line, indicating that the permeability shifts in response to ethanol are comparable to those associated with a shift in temperature. The majority of the mutants are situated on the plot closer to the origin than FBR5, indicating that those strains experience less pronounced changes in membrane permeability in response to environmental stimuli than the original FBR5 parent. Of particular note are the High Permeability mutants in that they have a constitutive increase in membrane permeability relative to FBR5 but are less responsive to environmental stimuli than FBR5.

As one might expect, the membrane permeability responses of the Low Permeability/Low MIC strains overlap with those of FBR5 (Fig. 4a). However, the data from this group of mutants are not clustered as tightly as the High Permeability strains. Thus, even though these strains do not show robust differences from FBR5 in the MIC assay, a number of them do exhibit differences in the membrane permeability assays. Finally, we note that although data from several of the Low Permeability/High MIC strains are similar, the strain ARR was found to be an extreme outlier with much greater permeability plasticity than any other strain in the collection.

To test whether the effects on membrane permeability induced by temperature or the addition of ethanol are additive, the relative effects of changing both variables were plotted against the addition of ethanol only (Fig. 4b) and against shifting the temperature only (Fig. 4c). Both of these comparisons resulted in graphs remarkably similar to each other and to Fig. 4a, suggesting that the variables of temperature and ethanol do not exert additive effects on membrane permeability. Consistent with this conclusion is the observation that adding ethanol to a 37 °C culture results in very little change in permeability, as does shifting a culture in ethanol from 30 °C to 37 °C. This is shown in Fig 4d and e, respectively, by the clustering of all strains near the y axis.

### Lipid profiling by GC-MS

In an effort to identify the underlying cause of differential permeability profiles, the lipid acyl chain composition of the strains was analyzed. As described in the Methods, lipids were extracted from cultures of *E.coli* grown under different temperature and ethanol conditions. These lipids were modified to form fatty acid methyl esters and subsequently analyzed by GC-MS. This analysis was non-quantitative, but simply used as a survey to identify any gross changes in the appearance or disappearance of membrane lipid constituents. The results of these assays for FBR5, ARR, and ARS strains are outlined in Table 4. In general, there were no major changes in the patterns of lipid acyl chains identified between strains. The major lipid acyl chains that were previously identified as present in the *E.coli* lipid membrane (both inner and outer membranes) [40–43] were found in all three strains, with some variation caused by growth temperature (30 °C vs. 37 °C) and the presence or absence of 4% EtOH in the growth media. Additional mutants from the collection, including strains from the High Permeability class and strains from the AN group, were analyzed and all of them yielded similar results (data not shown). These results suggest that the altered permeability exhibited by the mutant strains is mediated by a mechanism that does not involve changes in acyl chain constituents.

**Table 4:** Comparison of acyl chains detected in strains grown under different conditions^a^

Acyl Chain^b^	30 °C without Ethanol			30 °C with 4% v/v Ethanol			37 °C without Ethanol			37 °C with 4% v/v Ethanol		
	FBR5	ARR	ARS	FBR5	ARR	ARS	FBR5	ARR	ARS	FBR5	ARR	ARS
C12:0	+	+	+	+	+	+	+	+	+	+	+	+
C14:0	+	+	+	+	+	+	+	+	+	+	+	+
C14:1Δ7	n.d.	n.d.	n.d.	n.d.	n.d.	n.d.	n.d.	n.d.	n.d.	n.d.	n.d.	n.d.
C15:0	+	+	+	+	+	+	+	+	+	+	+	+
C16:0	+	n.d.	n.d.	+	+	n.d.	+	+	n.d.	n.d.	+	n.d.
C16:1Δ9	+	+	n.d.	+	+	n.d.	+	+	+	+	+	n.d.
C18:0	+	+	n.d.	+	+	n.d.	+	+	n.d.	n.d.	+	+
C18:1 Δ9	+	+	+	+	+	+	+	+	n.d.	+	+	+
C18:1 Δ11	n.d.	n.d.	+	n.d.	n.d.	n.d.	+	n.d.	+	n.d.	n.d.	n.d.

^a^Acyl chains compared to (ref. [40]). Abbreviations: +, lipid detected; n.d., not detected

^b^Acyl chains were isolated and converted to fatty acid methyl esters for analysis; see Methods

### Genomic sequence analysis of the mutant collection

To identify the genetic causes of altered membrane permeability and ethanol tolerance, the genomic sequences of all twenty mutants were obtained and compared to the genome of the parental FBR5 strain (Table 5). Each of the mutant strains contained only a small number of sequence changes (average of 1.7 mutations per strain) compared to FBR5. The maximum number of changes observed in a single strain was four (ANE, ANF, ANJ, ARN) and several strains (ANB, ANH, ARR) had no observed nucleotide changes (even when testing by two methods, as described in Methods). Of the latter group, three strains were categorized into the Low Permeability/Low MIC phenotype group, a group of strains with phenotypes very similar to FBR5. ARR, also in the latter group, was also devoid of mutations, even though ARR exhibits a high MIC phenotype and the greatest plasticity in membrane permeability. It is possible that ARR contains epigenetic changes that result in these extreme phenotypes. The observation that strains ANG, ANI, and ARO apparently have the same genomic sequence but belong to different phenotype categories is also suggestive that epigenetic mechanisms may be at work in at least some of the mutants in the collection. Another possibility is that undetected extrachromosomal sequences participate in these phenotypes. The genomic sequences of two strains, ANE and ANF, appear identical, suggesting that those two strains were not isolated independently. Supporting this idea, the two strains show similar MIC (Fig. 2) and permeability (Fig. 3) phenotypes.

**Table 5:** Mutations observed in ethanol tolerant strains derived from FBR5^a^

	Locus			High Permeability Strains^b^	Low Permeability Strains^c^	
Functional Category	Gene	Function	Mutation	High MIC	High MIC	Low MIC
Membrane Structure and Function						
	*xylG*	Component of a D-xylose ABC transporter	G67R	ARM*		
	*wza*	Outer membrane polysaccharide exporter	insertion at ~ 2,134,249 nt		ARS*	
	*waaG*	Catalyzes formation of D-glycosyl-lipopolysaccharide	S359R			ANE, ANF
	*yebZ*	Presumed inner membrane protein with unknown function	G164 *fs*			ANE, ANF
	*wbbH*	O antigen assembly	V97E			ARQ*
	*ynfB*	Presumed periplasmic protein, unknown function	V34 M			ANC
	*rybB*	sRNA regulating degradation of *omp* mRNAs in response to envelope stress^d^	P4T			ANC, AND
Ion Transport and Usage						
	*fecA*	Ferric citrate import^e^	V244 V	ARN, ARO*	ANG*	ANI*
	*yhiD*	Active transport of Mg^2+^ into the cell	T170A	ARN		
	*yfeX*	Iron retrieval from heme and PPIX	E151A			ANC, AND
Carbon Metabolism						
	*tdcE*	Pyruvate formate lyase	N26D	ARL*		
	*cdaR*	D-galactarate and D-glucarate metabolism	L151Q		ARK	
	*fdhF*	Formate dehydrogenase	V87F			ANA*
	*yfdE*	Oxalate CoA-transferase	K317 T			ANE, ANF
Respiration						
	intergenic between *torS/torT*	TMAO reductase	Insertion		ANJ	
	*ndh*	NADH deydrogenase II^f^	R306R			AND
Protein Synthesis and Related Functions						
	*valT,lysW,valZ*	tRNA genes	779,852–780,370 deletion	ARN		
	*rrlC*	Encodes 23S rRNA	Insertion at nt 3,943,588		ANJ	
Pyrimidine Metabolism						
	*carB*	Carbamoyl phosphate synthase	W213S	ARP		
	*preT*	Pyrimidine degradation	S32 T	ART		
	*ygeW*	Carbamoyl-transferase	D289V		ANJ	
Miscellaneous						
	*gyrA*	Gyrase; nalidixic acid resistance	R739C	ARN		
	*rhsD*	Inhibits growth of neighboring bacteria	T524A	ART		
	*rhsA*	Inhibits growth of neighboring bacteria	Insertion at nt 3,762,076 and deletion at nt 3,763,391		ANJ	
	*ytcA*	hypothetical protein	A49T		ANJ	
	*cheA*	Histidine kinase regulating flagellar rotation (chemotaxis)	P618L		ARK	
	many genes	deletion	excision of e14 prophage^g^			ANE, ANF
No mutations detected in ORFs					ARR	ANB, ANH

^a^Table lists mutations detected in open reading frames; for full list of all observed mutations, please see Additional File 1: Table S1. Abbreviations and symbols: *fs*, framsehsift; MIC, minimal inhibitory concentration; nt, nucleotide position; ORF, open reading frame; PPIX, protoporphyrin IX; TMAO, trimethylamine-N-oxide; *indicates strains for which only a single nucleotide alteration from FBR5 was observed

^b^These mutants display constitutively higher inner membrane permeability than FBR5 (see text and Table 3)

^c^These mutants display inner membrane permeability that is either similar to FBR5 or is higher than FBR5 but not constitutively (see text and Table 3)

^d^Various *omp* genes were implicated in ethanol stress response in [28]

^e^Previously implicated in ethanol stress response [28]

^f^Previously implicated in ethanol tolerance in [29]

^g^See refs. [69, 70]

In accordance with the GC-MS data, there were no changes in genes that directly control the synthesis or degradation of fatty acyl chains of inner membrane lipids. However, there were some gene changes in the strain collection that might affect cell permeability in other ways. For example, the High Permeability strain ARP contains a mutation in *ybbP*, an uncharacterized gene that encodes a member of the ABC transporter family [44]. Strain ARN carries a mutation in a putative magnesium transporter, *yhiD* [45] The ANC and AND strains carry a mutation in *rybB*, an sRNA gene that regulates the expression of outer membrane porin genes (reviewed in ref. [46, 47]). That said, a substantial number of strains in the collection do not carry any mutations in genes that are known to affect membrane permeability even though their permeability phenotypes differ significantly from those of FBR5 (Fig. 3). Examples include strains ARL, ARM, ARO, and ART at 30 °C without ethanol; strains ARS and ARR at 30 °C in the presence of ethanol; and strains ANA and ARK at 37 °C without ethanol.

Most striking was the observation that four strains (ANG, ANI, ARN, and ARO) carry the same C → A mutation in *fecA*, a gene that encodes a ferric citrate importer. Given that each of the 19 independent strains had an average number of 1.53 mutations, the probability of four strains randomly acquiring the same nucleotide change is 5.7 × 10^− 25^. For three of these strains, this was the sole mutation observed, further suggesting that this non-random mutation is linked to the altered ethanol and membrane phenotypes. Surprisingly, this DNA change results in a V244 V silent mutation, showing that there is no change in protein sequence. It is possible that this mutation affects translation or degradation of the transcript and thus protein levels.

### Importance of the *fecA* locus for survival in ethanol

**Fig. 5:**
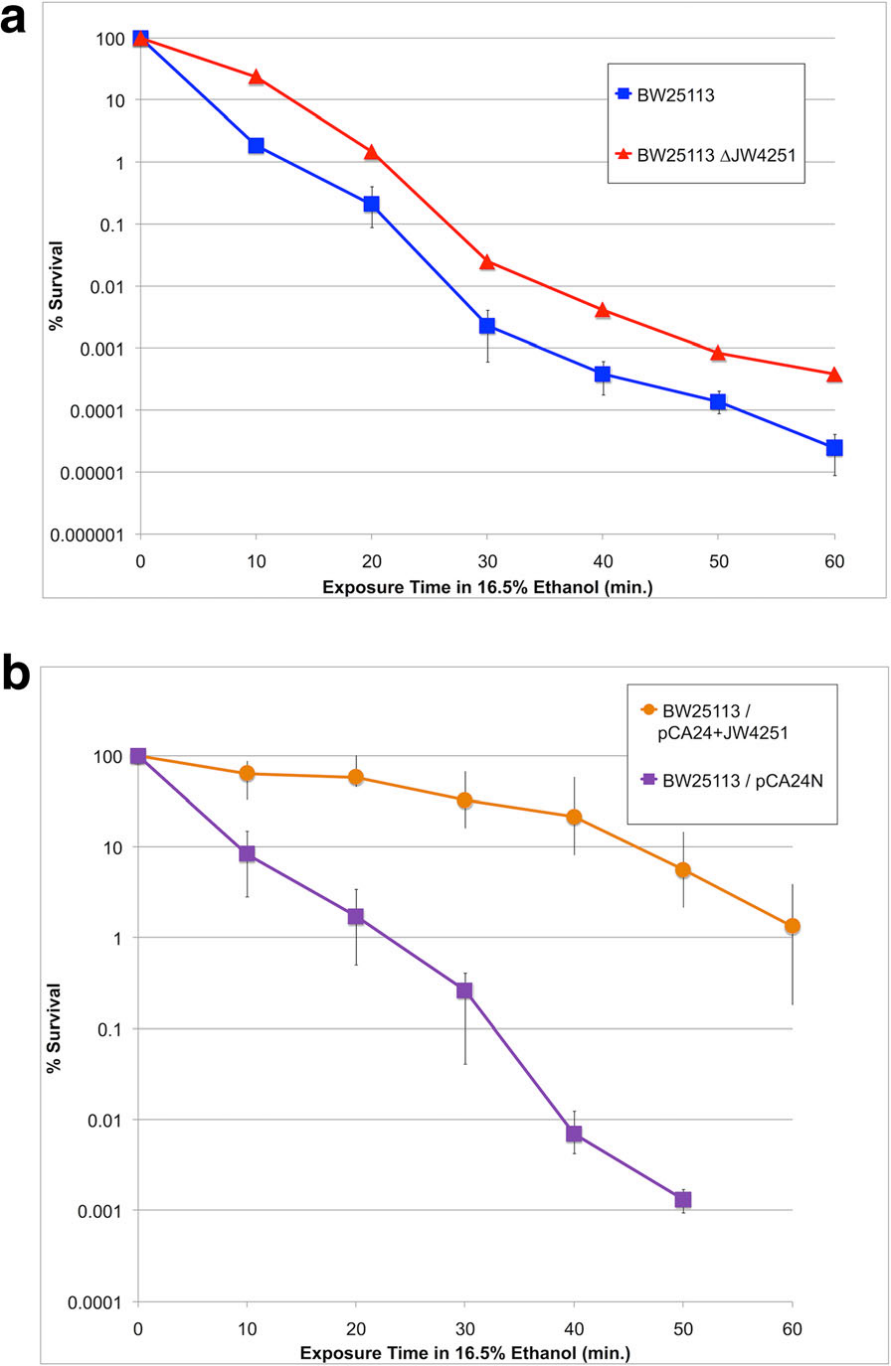
Survival curve data demonstrating the effect of *fecA* mutations on the ethanol tolerance phenotype of *E. coli.* Panel (**a)** depicts the performance of strains BW25113 and BW25113 ΔJW4251 (a *fecA* deletion strain) and Panel (**b**) depicts the performance of strains bMH33 and bMH34. Strains were grown for 60 min in 16.5% (*v*/v) ethanol, and viable cell counts were conducted at specific time points during that incubation period. In Panel (**b**), both cultures were also supplemented with 0.1 mM IPTG. For both panels, the data shown is the average of three technical replicates. Each of the plotted data points are the average of three independent trials. Error bars represent observed maximum and minimum values from all trials

To demonstrate that the *fecA* locus plays a causal role in ethanol tolerance, survival assays were carried out on BW25113 and its *ΔfecA* derivative BW25113 ΔJW4251. As shown in Fig. 5a, the *fecA* deletion strain exhibited approximately 10-fold greater survival during a 60 min challenge with 16% (*v*/v) ethanol. This result indicates that a loss of FecA activity increases the ethanol tolerance of *Escherichia coli*.

The effect of *fecA* over-expression was also examined using the plasmid pCA24N + JW4251, a construct bearing an isopropyl β-D-1-thiogalactopyranoside (IPTG)-inducible promoter fused to the *fecA* open reading frame. The pCA24N plasmid alone was used as a control. Fig. 5b shows the survival of BW25113 cells carrying these plasmids in the presence of IPTG and 16.5% (v/v) ethanol. The plasmid-only strain displayed significantly lower survival after an hour-long ethanol exposure as compared to the *fecA* over-expression strain. Thus, either a gain or a loss of *fecA* expression appears to improve the survival of *E. coli* in the presence of ethanol.

## Discussion

### FBR5 has greater ethanol sensitivity than the K-12 ancestor

We demonstrate here that the ethanologenic *E. coli* strain FBR5 is significantly more sensitive to ethanol than its ancestral K-12 strain (Fig. 1). This is somewhat unexpected as one might anticipate that FBR5 would have greater ethanol tolerance than *E. coli* K-12. Because ethanol fermentation is the only anaerobic metabolism available to FBR5, it would not be unreasonable to predict that normal culturing and passages of FBR5 in the laboratory would result in frequent exposure to ethanol, and thus also create selective pressure for the ability to grow in the presence of ethanol. Nevertheless, the fact remains that somewhere during its history the FBR5 lineage acquired ethanol sensitivity, a finding that is important with respect to the use of FBR5 as a ethanologenic biocatalyst.

The genome sequence of FBR5 includes mutations in several different loci that are known to experience substantial changes in expression in response to ethanol exposure or that have been implicated in ethanol tolerance (Table 2). Presumably these changes are the underlying cause for the ethanol sensitive phenotype of FBR5. Interestingly, FBR5 was reported as a xylose-utilizing mutant of NZN111 [4], but we did not observe any mutations in loci that are directly related to the metabolism of five carbon sugars. Mutations were observed in genes for a putative symport permease and two putative ABC transporters, however (Table 2). If one or more of those transporters is capable of moving five carbon sugars, then it is possible that the mechanism underlying the increased utilization of xylose by FBR5 is a change in the sugar transport activities rather than metabolic reactions inside the cell.

### Phenotypic characterization of ethanol tolerant mutants of FBR5

MIC experiments conducted during this study have shown FBR5 has an MIC below 4.5% *v*/v (Fig. 2), a value that is not dissimilar to the reported ethanol yield from FBR5 fermentation of corn fiber [3]. We report here the isolation of a collection of twenty FBR5 derivatives via a selective enrichment protocol, many of which have MIC values greater than 4.5% v/v (Fig. 2).

Alcohols have been shown to contribute to membrane leakage and resultant disruption of intracellular ion concentration which can lead directly to cell death [37–39]. This is consistent with in vitro and in silico results showing that ethanol disrupts acyl chain packing at the interior of the bilayer promoting the liquid disordered phase and can result in the formation of non-bilayer phases or interdigitated phases [48–52]. In addition to physical effects on the molecular behavior of membrane components, alterations in temperature can affect the rate of protein synthesis and other biochemical reactions key to survival and homeostasis [53]. It has been suggested that due to such effects, cellular maintenance of the membrane fluidity and permeability is a dynamic and tightly controlled process. It is known, for example, that environmental changes can induce alterations in membrane sterol content, in the ratio of desaturated to saturated acyl chains, and in the utilization of longer acyl chains in membrane lipids in various microorganisms [12, 54, 55]. *E. coli* is known to respond to temperature by regulating membrane fluidity through lengthening acyl chains and modulating the abundance of specific lipid species in the bilayer (cf. [55, 56]). *E. coli* has also been observed to alter its membrane constituents in response to ethanol (cf. [12, 37]), particularly in the presence of high concentrations of ethanol [48]. On the other hand, it has also been reported that the organism does not substantially alter its ratio of saturated and unsaturated lipids during ethanol exposure and this apparently allows its membrane fluidity to increase as a consequence [13].

For these reasons, it was important to examine the membrane permeability for each of the mutants under a variety of environmental conditions. Initial experiments measured indirectly ONPG diffusion across the inner membrane at 30 °C in the absence of ethanol, and these results (Fig. 3), in conjunction with MIC data (Fig. 2), facilitated categorization of the mutants in the collection (Table 3). Mutants with membrane permeability greater than FBR5, all of which also have an ethanol MIC greater than FBR5, were designated as High Permeability mutants. Mutants with membrane permeability comparable to FBR5 were denoted as Low Permeability mutants. Some Low Permeability mutants have MIC phenotypes greater than FBR5, and some do not.

As a group, the High Permeability/High MIC mutants show relatively little change in their membrane permeability in response to increases in temperature or ethanol concentration (Fig. 4). In other words, these strains maintain relatively high membrane permeability under all examined conditions and display considerably less dynamism in their membrane permeability than FBR5. Conversely, a number of Low Permeability/High MIC mutants show substantial changes in their membrane permeability phenotype in response to the environment (Fig. 4). For many of these strains, the magnitude of their phenotypic responses are roughly similar to that of FBR5. Thus, these strains have acquired higher ethanol tolerance than FBR5 but have maintained membrane permeability phenotypes that are similar to the parent strain. One mutant from the Low Permeability/High MIC group, ARR, is noteworthy in that it exhibits markedly greater changes in permeability than any other strain in the collection. Presumably strain ARR represents an ethanol tolerance mechanism that is somehow different from the rest of the Low Permeability/High MIC strains. Interestingly, the additive effects both ethanol exposure and temperature increase are apparently minimal in terms of the overall membrane permeability in all of our strains. Because the physiological responses of the membrane to temperature or ethanol do not appear to be additive, they may represent a threshold of adaptive response to environmental pressures.

A number of the Low Permeability/Low MIC strains appear to behave very similarly to FBR5 in the assays reported here. The abundance of low MIC strains in the collection likely means that the enrichment regimen used to isolate the mutant collection was not overly stringent, thus allowing strains similar to FBR5 to persist to the endpoint of the entire procedure. However, a number of the low MIC isolates do show substantially reduced plasticity in their membrane permeability relative to FBR5 (Fig. 4), thus confirming that they are physiologically distinct from FBR5. Consequently it may be that these strains may exhibit greater ethanol tolerance than FBR5 under conditions that are more similar to those of the enrichment protocol rather than the MIC assays, although this is currently untested.

To further investigate the permeability shifts, we performed a series of qualitative analyses on membrane lipid composition of the strains. We have examined the lipid composition of a number of representative strains from our collection and detected no gross changes in membrane constituents relative to FBR5 in response to a temperature shift or to a 4% *v*/v ethanol challenge (Table 4). Thus, ethanol tolerance in all of our high MIC strains likely result from a mechanism other than membrane lipid metabolism, a result that is concordant with the observations reported for K-12 and MG1655 by Huffer et al. [13]. In addition, the increased membrane permeability of the High Permeability strains and their ability to maintain that permeability constitutively must also arise via a mechanism other than membrane lipid metabolism.

### Mutational changes in the ethanol tolerant strain collection

Beyond membrane lipid metabolism, published literature regarding ethanol tolerant *E. coli* and other ethanologenic microbes suggests that, in principle, alterations in sugar transport, increased TCA activity, transcriptional regulation of electron transport components or fermentation enzymes, altered expression of transcriptional regulators such as FNR, increased peptidoglycan synthesis, elevated biosynthesis or transport of various amino acids, increased betaine production, and uptake or retention of metals such as iron or zinc can contribute to ethanol tolerance (cf. [28, 29, 57–59]) reviewed in [60]. Indeed, changes to genes in a number of those categories are represented in our collection of ethanol tolerant strains. Consistent with the GC-MS results, we observed no mutations in known lipid biosynthetic genes or in known regulators of membrane lipid and porin genes such as *invR*, *micA*, *omrA/B*, or *rseX* (reviewed in [46, 47, 61]). Only two strains (ANC, AND) carry a change in *rybB*, a regulator of outer membrane porin genes (reviewed in [46, 47]).

None of the mutants display extensive genetic changes. This may be the consequence of using sub-lethal concentrations of alcohol during the mutant isolation procedure. Remarkably, some members of the mutant collection do not have detectable nucleotide changes, including the Low Permeability/High MIC outlier ARR. In addition, three strains are apparently genetically identical (ARO, ANG, ANI) but their performance in our experiments merited placement into separate phenotypic categories. Taken together, these observations suggest that epigenetic mechanisms may be at work in at least some members of the collection regardless of the number of detected nucleotide changes. We note that another study [62] has also suggested that epigenetic mechanisms can contribute to ethanol tolerance in *E. coli*.

### Evidence that *fecA* can influence ethanol tolerance

The most surprising observation from the genomic data of the mutant collection was that four of the strains independently acquired an identical single nucleotide change in the *fecA* gene (Table 5). FecA is an outer membrane protein that is required for iron acquisition via ferric citrate uptake [63]. Survival assays demonstrated that either the deletion (Fig. 5a) or the over-expression (Fig. 5b) of *fecA* in BW25113 improves ethanol tolerance. Although changes in *fecA* transcription have been implicated in the *E. coli* ethanol stress response of *E. coli* [28], to the best of our knowledge, this is the first direct phenotypic demonstration of an association between the *fecA* genotype and ethanol tolerance.

Importantly, the recurring mutation in *fecA* is translationally silent, and the predicted amino acid structure of the mutant FecA protein is not expected to be different from that of the parental FBR5 strain. It is possible that the location of the silent mutation observed in our mutants may be involved in some type of RNA-RNA interaction that serves to regulate *fecA* expression. If this is the case, then the observation that same nucleotide change appeared in four independent lineages would be suggestive that this particular nucleotide plays a critical role in the hypothesized RNA base pairing. In *E. coli*, iron uptake and metabolism is regulated in part by a small RNA molecule RyhB [64, 65] and it has been shown that *fecA* is weakly regulated by RyhB [65]. Whether the RNA-mediated regulation that we propose as an explanation for the importance of the silent *fecA* mutation described here is related to RyhB activity or is a consequence of some other molecule remains to be seen.

The apparent benefit to cell survival when *fecA* is deleted or over-expressed makes it difficult to predict whether the observed silent mutation confers an increase or decrease in FecA synthesis. In addition, an important caveat to the experiments reported here is that the *kan* insertion marker in strain BW25113 Δ JW4251 is likely to be polar and alter expression of the entire *fec* operon. In contrast, the pCA24N + JW4251 plasmid expresses the *fecA* open reading frame rather than the entire *fec* operon. Nevertheless, our results collectively suggest that the levels of expression from the *fecA* locus are important to the *E. coli* cell when it is exposed to ethanol.

## Conclusion

We describe here the characterization of a collection of ethanol tolerant strains derived from the ethanologenic *Escherichia coli* strain FBR5. Many of the strains in the collection have membrane permeability alterations, but GC-MS revealed no qualitative changes in the acyl chain components of their membrane lipids. None of the strains displayed mutations in genes known to control membrane lipid synthesis, and in fact a few strains carried no mutations at all. It was also observed that four of the strains acquired an identical C → A (V244V) silent mutation in the ferric citrate transporter gene *fecA*. We present the first direct phenotypic evidence that changes in the expression of *fecA* can influence ethanol tolerance. We suggest that the recurring silent mutation might alter RNA-mediated regulation of *fecA* expression.

## Additional file

Additional file 1: **Table S1.** List of observed nucleotide changes in FBR5 and the twenty mutant strains derived from FBR5. *Escherichia coli* K-12 MG1655 (GenBank: U00096.2) was used as the reference genome. (XLSX 22 kb)

## Abbreviations

Ap: Ampicillin
Cm: Chloramphenicol
CTAB: Cetyl trimethylammonium bromide
del: Deletion
*E. coli*: *Escherichia coli*
fs: Frameshift
GC-MS: Gas chromatography mass spectrometry
IGV: Integrated genome viewer
IPTG: Isopropyl β-D-1-thiogalactopyranoside
Kan: Kanamycin
LB: Luria Bertani
MIC: Minimal inhibitory concentration
n.d.: Not detected
nt: Nucleotide
ONP: O-nitrophenol
ONPG: O-nitrophenylgalactopyranoside
ORF: Open reading frame
PBS: Phosphate buffer solution
PPIX: Protoporphyrin IX
Tc: Tetracycline
TMAO: Trimethylamine-N-oxide
